# Application of paclobutrazol affect maize grain yield by regulating root morphological and physiological characteristics under a semi-arid region

**DOI:** 10.1038/s41598-018-23166-z

**Published:** 2018-03-19

**Authors:** Muhammad Kamran, Su Wennan, Irshad Ahmad, Meng Xiangping, Cui Wenwen, Zhang Xudong, Mou Siwei, Aaqil Khan, Han Qingfang, Liu Tiening

**Affiliations:** 1 College of Agronomy, Northwest A&F University/Key Laboratory of Crop Physio-ecology and Tillage in Northwestern loess Plateau, Ministry of Agriculture, Yangling, Shaanxi 712100 China; 2 Institute of Water Saving Agriculture in Arid Areas of China, Northwest A&F University, Yangling, Shaanxi 712100 China; 3 College of Agronomy, Anhui agricultural university, Hefei, 230036 China

## Abstract

A field experiment was conducted to investigate the effects of paclobutrazol on ear characteristics and grain yield by regulating root growth and root-bleeding sap of maize crop. Seed-soaking at rate of 0 (CK1), 200 (S1), 300 (S2), and 400 (S3) mg L^−1^, and seed-dressing at rate of 0 (CK2), 1.5 (D1), 2.5 (D2), and 3.5 (D3) g kg^−1^ were used. Our results showed that paclobutrazol improved the ear characteristics and grain yield, and were consistently higher than control during 2015–2016. The average grain yield of S1, S2 and S3 were 18.9%, 61.3%, and 45.9% higher, while for D1, D2 and D3 were 20.2%, 33.3%, and 45.2%, compared to CK, respectively. Moreover, paclobutrazol-treated maize had improved root-length density (RLD), root-surface area density (RSD) and root-weight density (RWD) at most of the soil profiles (0–70 cm for seed-soaking, 0–60 cm for seed-dressing) and was attributed to enhancing the grain yield. In addition, root-activity, root-bleeding sap, root dry weight, diameter and root/shoot ratio increased by paclobutrazol, with highest values achieved in S2 and D3 treatments, across the whole growth stages in 2015–2016. Our results suggested that paclobutrazol could efficiently be used to enhance root-physiological and morphological characteristics, resulting in higher grain yield.

## Introduction

Maize (*Zea mays* L.) is one of the most important staple crops worldwide with multiple uses which include food, fodder, industrial material, and bioenergy. China is among the major maize producer countries in the world, with total maize area of approximately 36 million ha, and the total maize production of more than 218 million tons in 2013^1,2^. However, the majority of the maize cultivated area is almost wholly rain-fed covered in the semi-arid area of the northwestern Loess Plateau. These agro-ecological regions account for about 56% of the nation’s total cultivated land^3^, and are characterized by low, erratic rainfall and periodic droughts^2^. Usually, a maize crop requires about 500–800 mm of water during its lifespan^4^. However, the limited and erratic rainfall during the growth period of crops are the major constraints which often leads to water stress, consequently resulting in drought^5,6^, and thereby reduces grain yield by about 50% and over^7^. Conversely, the world population is burgeoning and estimated to be 9.1 billion by 2050, thus necessitating the projected 70% increase in demand for agricultural production^8^. Fulfilling the food requirements for an ever-growing population is a major challenge of food security, when there are no new available areas for agricultural production and the existing ones are degrading by abiotic stresses, majorly drought stress. Part of the solution is to increase crop tolerance to water deficit condition through extensive root systems of maize in the dryland agricultural farming.

Roots are the prime organ of plants in terrestrial ecosystems, and knowledge on root morphology is essential for optimizing maize production especially in the semiarid areas, which determines the uptake and utilization of water and nutrients^9^. Root morphology can significantly influence the capacity of water extraction and nutrient uptake in crop plants^10^. Hence, the crop growth and final grain yield are substantially dependent on the root spread and distributions in any soil profile^11,12^. Root distribution and extension could be expressed as root length density (RLD), root surface area density (RSD) or root weight density (RWD)^11,13^. Roots with greater RLD and RSD can increase nutrient supply to the plant than those with shorter roots^9^. For example, roots with a higher surface area absorb a higher amount of nitrate and phosphorus^14,15^. An extensive rooting system is vital when plants are grown in soils containing insufficient supplies of nutrients or water^16^. At earlier growth periods, development of a more extensive root system could better adapt the crop to the semiarid environments^10^. The root-bleeding sap is another essential root characteristics and is the manifestation of root pressure, and its quantity reveals the potential growth and activity of root^9^. The bleeding sap is consistent with root activity, are helpful to understand the root behavior in field trials^17,18^. The various root traits have a direct impact on drought resistance of crops, more prominently under rainfed areas^12^. Understanding of key root traits and their response to environmental stimuli are crucial for optimizing plant growth and productivity, especially under limited water resources^16,19^.

To cope with water stress and to increase the adaptability of plants in the rainfed dryland region, the possible solution is either to manage the crop or otherwise to improve its genetic makeup. Although genetic engineering has helped in promoting the drought tolerance of crops^20^, however, previous literature has raised negative opinion suggesting that genetic makeup is not the specific and rapid method for drought resistance, which has triggered a debate^21^. In lieu of genetic engineering, exogenous application of plant growth regulators (PGRs) has emerged as an alternative approach for improving drought tolerance in plants, without altering its genetic makeup^20,22–24^. Paclobutrazol, a triazole plant growth regulator, have been extensively used in horticultural crops, majorly for producing shorter plant canopies, and its anti-gibberellic behavior has been well documented in plants^25–27^. Paclobutrazol inhibits gibberellic acid (GA) and endogenous indole acetic acid (IAA), while stimulates abscisic acid (ABA), cytokinin and ethylene concentrations within the plants^28,29^. Previous investigations have reported a crucial role of auxins and cytokinins in promoting growth and development of lateral and adventitious root^30,31^. Nevertheless, the literature has shown the plentiful efficacy of paclobutrazol and certain other triazoles for enhancing proline and soluble proteins, lignin content, reducing transpiration rate through the partial closure of stomata in various crops^25,27,31,32^. Previous research studies have suggested a potential role of paclobutrazol in improving the drought tolerance of crop plants by inducing antioxidant enzyme activities. However, the lack of in-depth understanding of effects of paclobutrazol on morphological and physiological characteristics of roots in field crops under water deficit condition limits their application in crop plants.

The literature regarding the potential effects of paclobutrazol on root growth characteristics including, root activity, root diameter, RLD, RWD, RSD, and rate of root bleeding sap flow is limited, and the relationships of root growth and grain yield under water deficit condition have not been investigated in details. Accordingly, the present trial was conducted to study the possible effects of seed-soaking and seed-dressing with different concentrations of paclobutrazol on the root morphological and physiological characteristics and investigate the relationship between improvement in root growth and increase in final grain yield of maize under a semi-arid water deficit condition^4^.

## Results

### Root activity and root-bleeding sap flow

**Figure 1:**
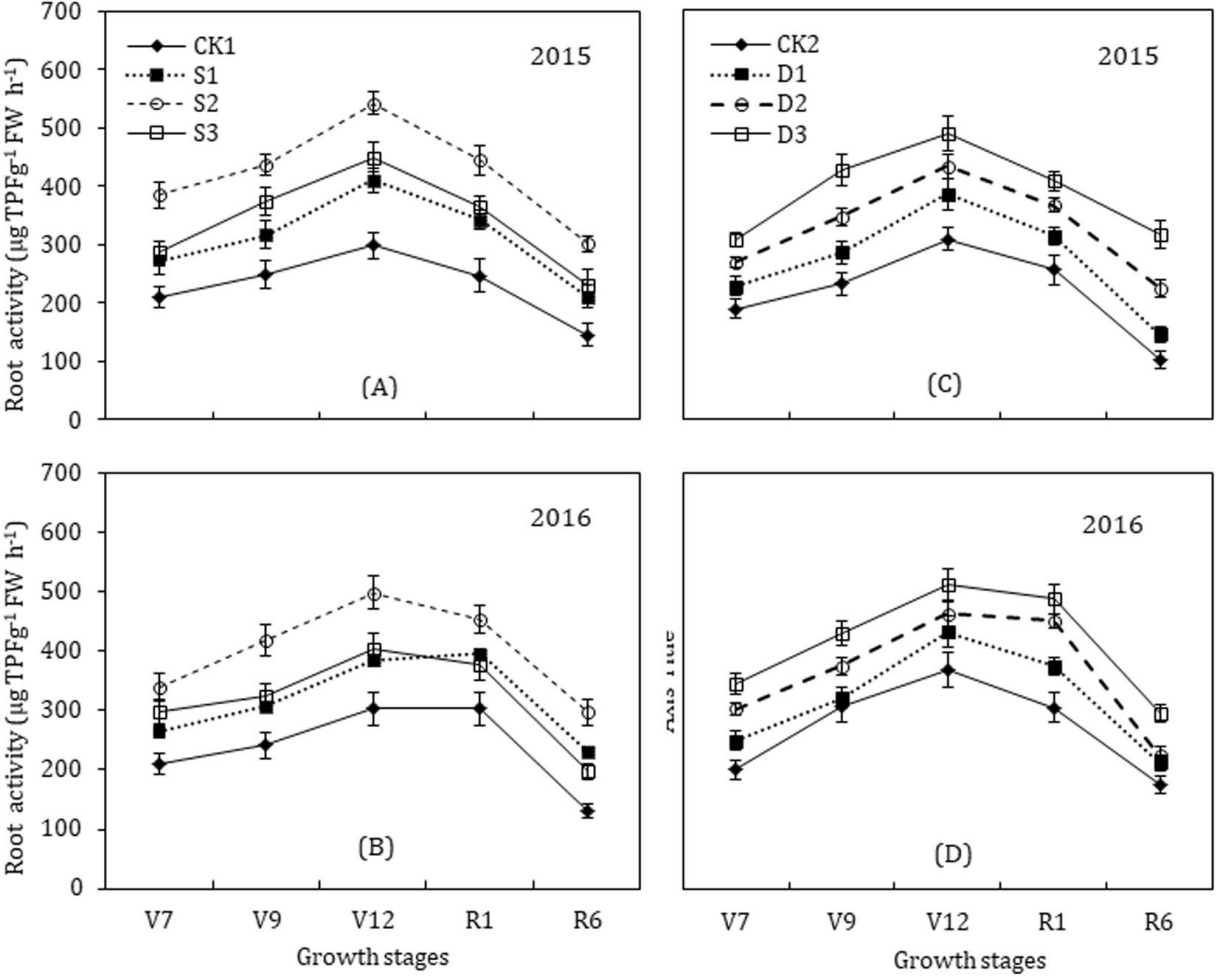
Effects of seed soaking (**A**, **B**) and seed dressing (**C**, **D**) with paclobutrazol on the dynamic changes in root activity of maize. The vertical bars represents mean ± S.D. (n = 3).

In the present study, paclobutrazol application both as seed-soaking and seed-dressing showed a marked influence on the quantities of triphenyl tetrazolium chloride (TTC) in roots, as a measure of root vigor (Fig. 1). Root TTC activities of maize crop increased gradually in the early growth stages and reached to single peak curves at the twelfth leaf stage (V12), and then declined in the late growth stages up to physiological maturity (R6) in all treatments. The paclobutrazol treated plants have significantly (*P* < 0.05) higher root TTC activity compared with untreated control plants (CK). The paclobutrazol increased the root activity in a dose-dependent manner; however, the highest concentration of paclobutrazol in seed soaking (S3, 400 mg L^−1^) inhibited the root activity and was lower than that in S2 treatment (300 mg L^−1^). No significant differences were observed for root TTC activities of S1 (200 mg L^−1^) and S3 (400 mg L^−1^) plants after the silking stage (R1). The highest activity was observed in roots of S2, and D3 (3.5 g paclobutrazol kg^−1^ seeds) treated plants. Root activity of S2 treated plant was 73.2% greater at seventh leaf stage (V7), 73.5% at ninth leaf stage (V9), 73.2% at twelfth leaf stage (V12), 63.4% at silking (R1), and 116.2% greater at the physiological maturity (R6) stage, than those in CK1 plants, respectively. Compared to CK2 plants, roots activity of D3 treated maize plants were 67.3% greater at V7, 59.0% at V9, 47.8% at V12, 59.5% at R1, and 119.1% greater at R6 stage, respectively.

**Figure 2:**
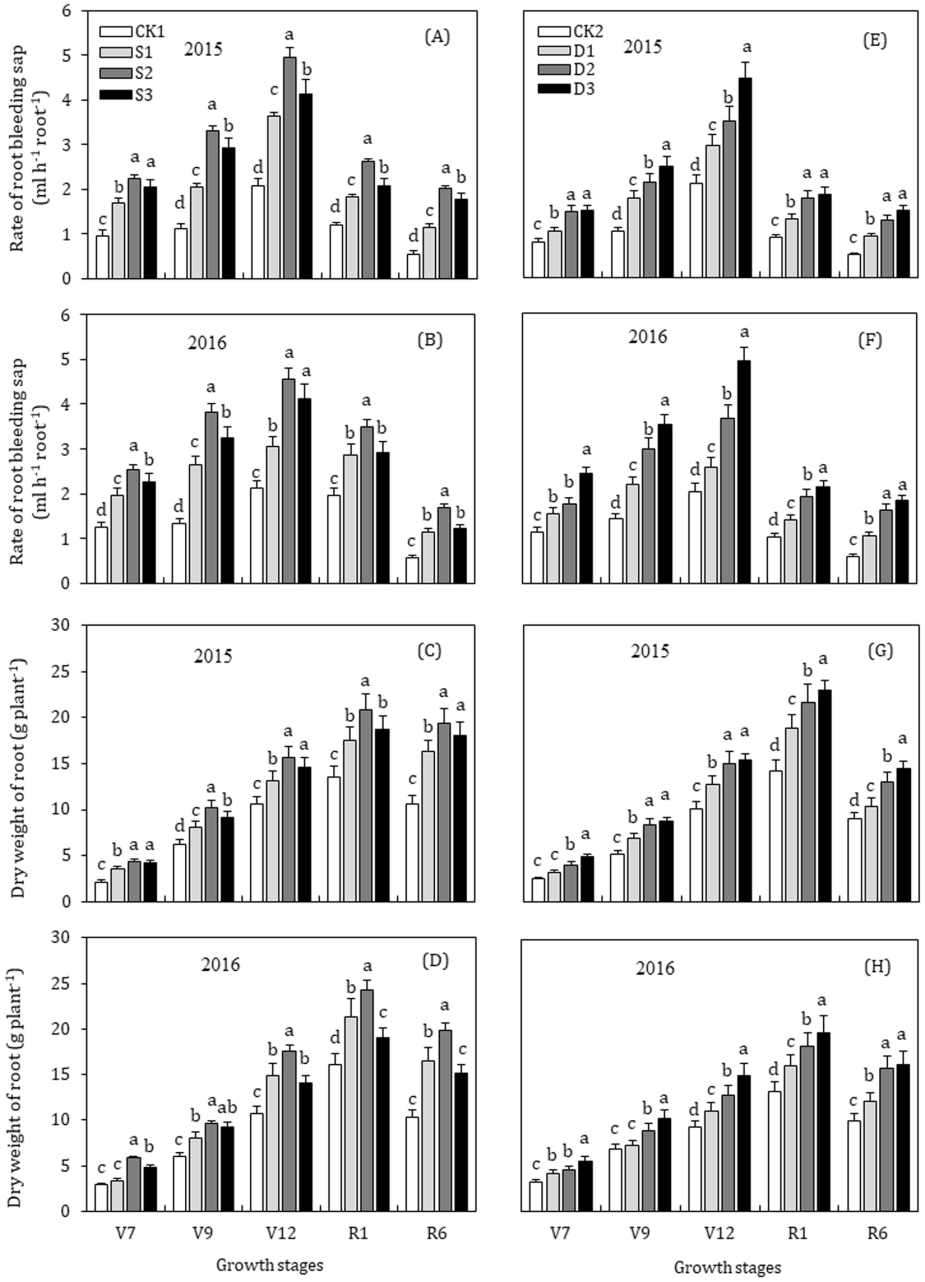
Effects of  seed soaking (**A**, **B**, **C**, **D**) and seed dressing (**E**, **F**, **G**, **H**) with paclobutrazol on the dynamic changes in root-bleeding sap rate and root dry weight of maize. The vertical bars represent mean ± S.D. (n = 3). Means followed by different letters within each growth stage represent significant differences (*p* < 0.05).

Paclobutrazol application revealed a significant effect (*P* < 0.05) on root-bleeding sap flow of maize through the whole growth seasons of maize crop during 2015–2016 (Fig. 2). The values of root-bleeding sap flow reached to a maximum at the V12 stage and then declined gradually in the later stages in all treatments. The rate of root bleeding sap flow increased in a dose-dependent manner with the concentration of paclobutrazol, where S2 and D3 treatments had higher values for root-bleeding sap flow, compared to control plants. No further significant increase was observed at a concentration higher than S2 in seed-soaking and root-bleeding sap flow was dramatically lower in S3 treated plants. Root-sap flow exhibited a similar varied trend during 2015–2016. The mean rate of root-bleeding sap flow across the two years of S2 treatment was higher by 116.0%, 191.7%, 126.5%, 91.4% and 230.5%, at V7, V9, V12, R1, and R6 stages, as compared with CK1, while that of D3 treatment was maximum at 100.6%, 144.1%, 123.5%, 108.7% and 197.9%, as compared with CK2 treatment, respectively. The increase in root activity and root-bleeding sap flow for each of the paclobutrazol treatments in seed soaking was ranked as S2 (300 mg L^−1^) > S3 (400 mg L^−1^) ≥S1 (200 mg L^−1^) > CK1 (control); while for seed-dressing treatments was D3 (3.5 g kg^−1^) > D2 (2.5 g kg^−1^) > D1 (1.5 g kg^−1^) > CK2 (control).

### Root diameter (cm)

**Figure 3:**
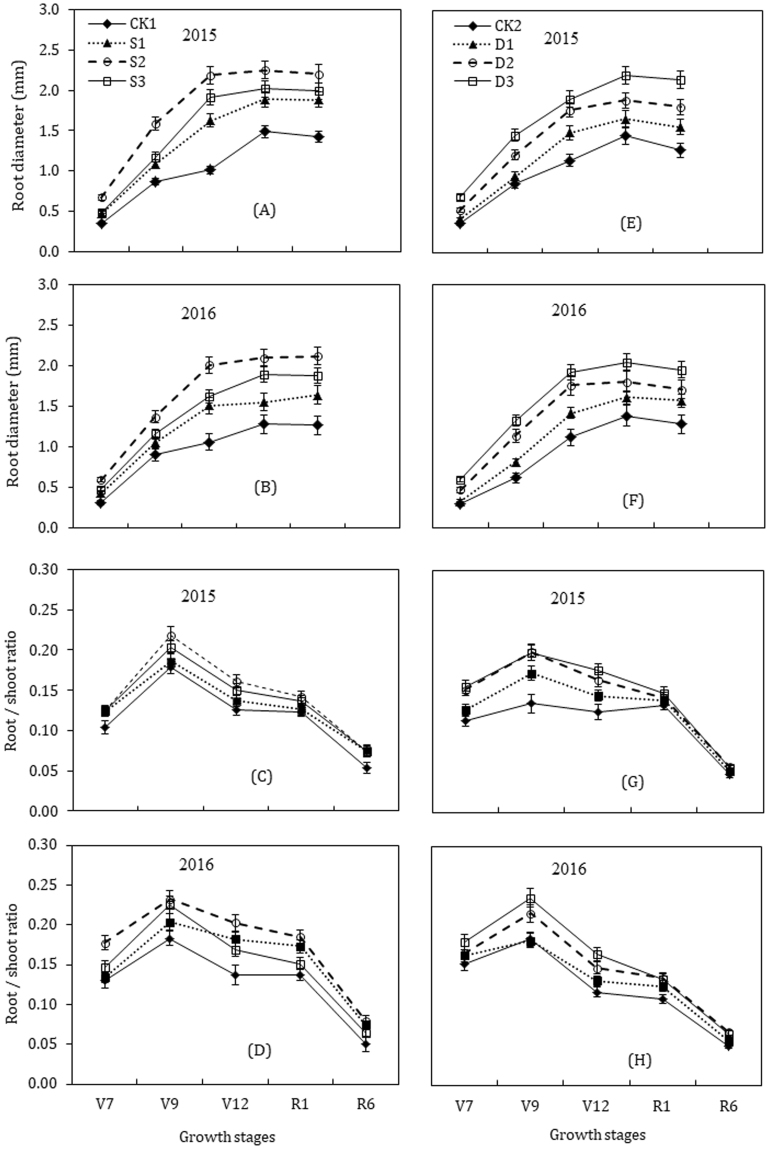
Effects of seed soaking (**A**, **B**, **C**, **D**) and seed dressing (**E**, **F**, **G**, **H**) with paclobutrazol on the root diameter and root to shoot ratio of maize. The vertical bars represent mean ± S.D. (n = 3).

Paclobutrazol treatments had a strong influence on root diameter during the successive growth stages, which initially increased with the increase of the concentration of paclobutrazol and then decreased at the highest concentration (S3) in seed-soaking, while linearly increased under seed-dressing (Fig. 3). The root diameter increased progressively from V7 to V9 stage, rapidly from V9 to V12 growth stage, and decreased gradually in the late growth stages in all treatments. When compared to CK1, the average root diameter of maize plants across the two growing seasons in S2 treatment was greater by 92.7% at V7, 66.9% at V9, 102.7% at V12, 57.3% at R1 and 60.7% higher at the R6 stage, respectively. Compared with CK2, the root diameter of maize plants under D3 treatment was greater by 92.4% at V7, 88.8% at V9, 68.7% at V12, 50.0% at R1 and 60.4% higher at the R6 stage, respectively. The average root diameter across the two years under seed-soaking treatments were in the order of; S2 > S3 ≥ S1 > CK1; while for seed-dressing treatments were; D3 > D2 > D1 > CK2.

### Root dry weight and root to shoot ratio

The dynamic changes in root dry weight and root/shoot ratio throughout maize developmental stages are presented in Figs 2 and 3. Root dry weight (g plant^−1^) of maize at successive growth stages varied considerably in paclobutrazol treatments in 2015 and 2016 (Fig. 2). The root weight increased in a dose-dependent manner with paclobutrazol, but the highest concentration of paclobutrazol under seed-soaking (S3) reduced the dry weight. In 2015, root dry weight of S2 and D3 treatments were increased by 102.1% and 95.5% at the V7 stage, 65.1% and 70.2% at V9, 47.9% and 52.1% at V12 stage, compared to CK, respectively. The root dry weight reached to a maximum value at R1 stage, where S2 treatment had the highest value of 20.9 g plant^−1^ (53.6% greater than CK1) among seed-soaking treatments, and that of D3 was 22.9 g plant^−1^ (61.3% higher than CK2) in seed-dressing. At R6 stage, root dry weight values decreased gradually and for S2 and D3 were 82.3% and 61.5% greater than control treatments, respectively. In 2016, root dry weight exhibited a similar trend, except for S3 in seed-soaking, which dramatically inhibited root dry weight and was considerably lower than S1 treatment at R1 and R6 stages. The dry weight of roots under S2 treatment was greater by 102.4%, 60.8%, 64.9%, 51.4% and 92.0%, at V7, V9, V12, R1 and R6 stage, while that of D3 was 69.9%, 49.7%, 61.7%, 49.7% and 62.5%, greater than untreated control, respectively.

The root/shoot ratio increased with plant growth, reached to a maximum at the V9 stage, and subsequently decreased to the R6 stage (Fig. 3). In seed-soaking treatments, the root/shoot ratio was significantly higher in S2 and S3 treatments and was markedly similar at various growth stages in 2015. In 2016, the root/shoot ratio was decreased dramatically in S3 treatment and was even smaller than that in S1 treatment from V12 to R6 stage. Compared with CK1, the mean root/shoot ratio of S2 was 28.8%, 24.1%, 38.5%, 25.2% and 47.7% higher at V7, V9, V12, R1 and R6 stage, respectively. In seed-dressing, the root/shoot ratio significantly increased with the increase of the concentration of paclobutrazol and was significantly higher in D3 followed by D2 throughout the growing season. Compared to that of CK2, the mean root/shoot ratio in D3 treatment was higher by 26.5%, 36.2%, 41.0%, 16.8% and 26.0% at V7, V9, V12, R1 and R6 stages, respectively.

### Root weight density (RWD)

**Figure 4:**
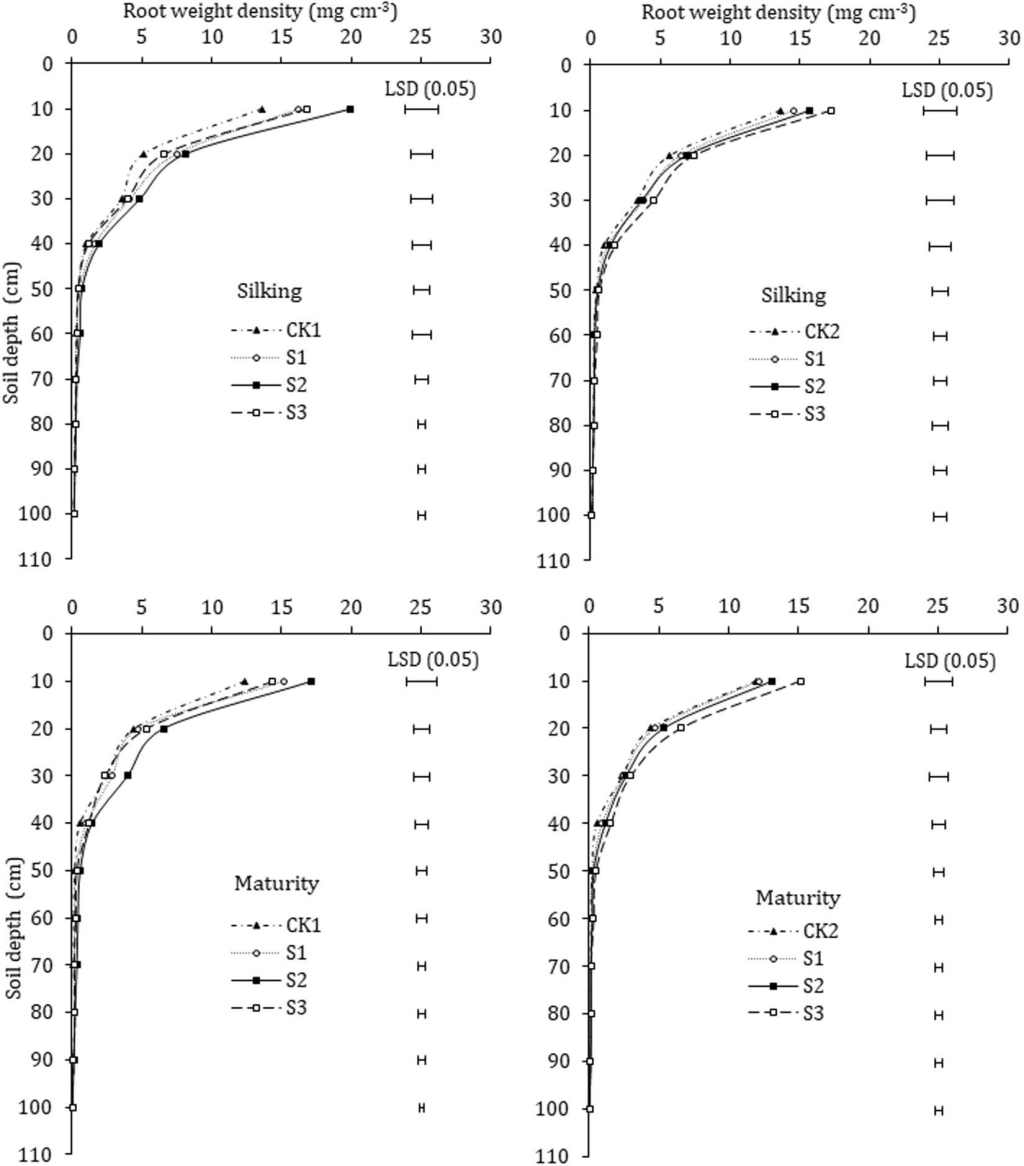
Effects of paclobutrazol application on the root weight density (RWD) of maize. Vertical bars represent the LSD (P < 0.05) for mean comparison between treatments.

Root weight density (RWD) is one of the essential parameter used for the evaluation of roots morphology. Roots of paclobutrazol treated maize crop exhibited statistically different growth patterns across 0–100 cm soil profile. The root weight density (RWD) was significantly (*P* < 0.05) higher in all paclobutrazol treated plants than untreated control plants for most of the soil depths (from 0–100 cm soil profile, with 10 cm increment each) (Fig. 4). Under seed soaking, at silking and maturity, RWD in 0–80 cm soil profile showed evident differences among paclobutrazol treatments and was significantly greater for S2 treatment (except for 90–100 cm). At silking, RWD under S2 treatment was markedly greater, and was increased by 47.2%, 58.5%, 38.0%, 88.1%, 48.7%, 89.2%, 23.2%, 43.9%, and 16.8% in 0–10, 10–20, 20–30, 30–40, 40–50, 50–60, 60–70, 70–80 and 80–90 cm soil profile, while greater by 38.9%, 47.3%, 57.3%, 151.8%, 238.6%, 259.5%, 217.8%, 26.0% and 29.5% at maturity, compared to that under CK1, respectively. In seed-dressing, greater RWD was exhibited by D3 treated plants and the differences were more evident in the 0–60 cm soil profile. RWD under D3 was greater at 27.1%, 30.5%, 36.2%, 81.6%, 52.9%, 116.1% in 0–10, 10–20, 20–30, 30–40, 40–50, and 50–60 cm soil profile at silking, while greater by 26.9%, 47.3%, 25.6%, 189.1%, 168.6%, 175.6% at physiological maturity, compared to that under CK2, respectively. There was no evident difference in RWD of paclobutrazol treatments and the untreated control in 60–100 cm soil profile under seed-dressing.

### Root length density (RLD) and root surface area density (RSD)

**Figure 5:**
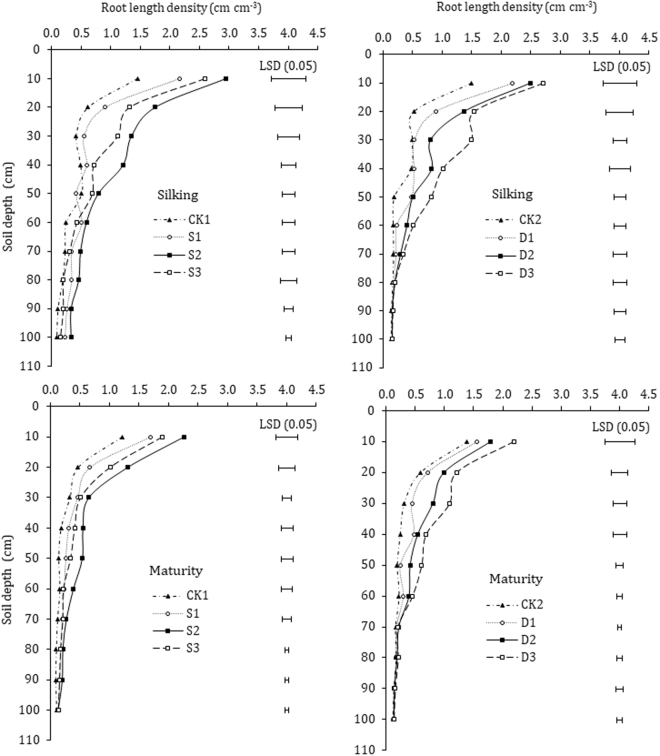
Effects of paclobutrazol application on the root length density (RLD) of maize. Vertical bars represent the LSD (P < 0.05) for mean comparison between treatments.

Root length density (RLD) is an essential determinant of crop nutrient and water acquisition. The maize roots grew gradually downward with the crop growth and reduced from silking to maturity stage across the 0–100 cm soil profile (Fig. 5). At silking and physiological maturity, RLD in most of the soil profile exhibited clear differences among different treatments under seed soaking and dressing . The highest concentration of paclobutrazol (S3) in seed soaking dramatically inhibited RLD in 0–50 cm soil profile, and was significantly smaller than S1, but was higher than control treatment. Statistically, there was no significant (*P* > 0.05) difference in RLD for seed-soaking treatments in 80–100 cm soil profile depths, and 60–100 cm for seed-dressing treatments at silking and maturity. At silking stage, mean values of RLD for S2 treatment were 103.1%, 182.3%, 221.4%, 146.9%, 54.9%, 136.0%, 113.0%, and 142.1%, higher, while, at physiological maturity were 86.8%, 178.7%, 97.0%, 205.6%, 285.7,%, 143.8%, 125.0%, and 110.0% higher than that of CK1 treatment in the 0–10, 10–20, 20–30, 30–40, 40–50, 50–60, 60–70, and 70–80 cm soil profile depths, respectively. The RLD under D3 treatment was 81.9%, 196.5%, 202.8%, 111.7%, 327.4%, 200.6% and 200.6% higher in 0–70 cm soil profile at silking stage, while 57.6%, 105.1%, 251.6%, 186.1%, 221.1%, and 106.1% higher at physiological maturity, compared with that of CK2 in 0–60 cm soil profile with each 10 cm increment, respectively.

**Figure 6:**
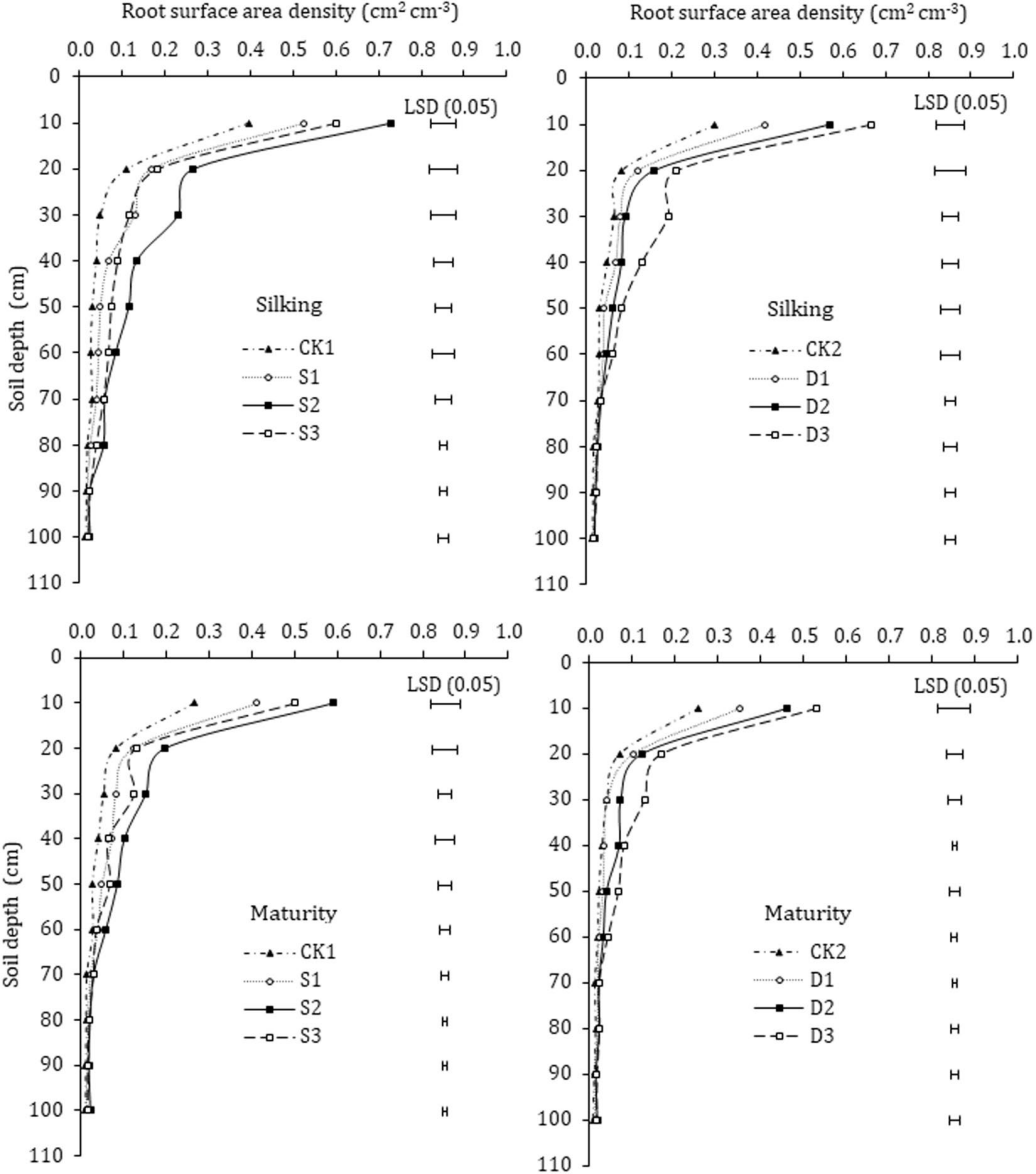
Effects of paclobutrazol application on the root surface area density (RSD) of maize. Vertical bars represent the LSD (P < 0.05) for mean comparison between treatments.

Root surface area density (RSD) under different paclobutrazol treatments exhibited a similar varied trend for RSD in the 0–100 cm soil depths under seed-soaking and dressing (Fig. 6). The paclobutrazol treatments showed significantly (P < 0.05) greater RSD than untreated control plants at different growth stages. In seed-soaking RSD indicated clear differences among different paclobutrazol and control treatments in 0–80 cm soil profile, but the highest RSD was observed under S2 followed by S3 treatment, as compared with untreated control (CK1), and there were no apparent differences found between S3 and S1 treatments at various soil depths. In seed dressing, paclobutrazol treatments showed significant differences mostly in 0–70 cm soil profile. However, there were no statistically significant differences in RSD of 70–100 cm soil profile depths among different treatments at silking and RSD of 60–100 cm soil profile at maturity stage. The RSD of different treatments under seed soaking was significantly higher in order of S2; S3; S1 across the (0–100 cm) soil profile depths at silking and physiological maturity, while for seed dressing were D3; D2; D1, as compared with CK1 and CK2 treatments (Fig. 6).

### Ear characteristics and grain yield

The results of field experiments in 2015–2016 showed that paclobutrazol treatments significantly (P < 0.05) enhanced the ear characteristics (ear length and diameter, kernels ear^−1^, and 1000 kernel weight) and grain yield of summer maize when compared with control treatments. During 2015–2016, thousand kernel weight (TKW), kernels ear^−1^, and grain yield were all significantly increased with the paclobutrazol application (Table 1). Compared with CK, S2 and D3 treatments had the best effects on the yield, followed by S3 and D2. Nevertheless, the highest concentration of paclobutrazol in seed soaking, i.e., S3 had a relatively small impact on increasing yield, and had inhibitive effects but compared with control treatment (CK), the grain yield was still improved by a relatively large amount. The two year average grain yields under S1, S2, and S3 treatments were increased by 1214.1 kg ha^−1^, 3943.3 kg ha^−1^, 2950.7 kg ha^−1^, respectively, i.e. 18.9%, 61.3%, and 45.9% increases compared with CK1, whereas that of D1, D2 and D3 increased by 1240.0 kg ha^−1^, 2044.8 kg ha^−1^, 2775.5 kg ha^−1^, respectively, i.e., 20.2%, 33.3%, and 45.2% increases compared with CK2.

**Table 1:** Effects of paclobutrazol on ear length (cm), ear diameter (mm), kernels ear^−1^, thousand grain weight (g), and grain yield (t ha^−1^) of maize in 2015–2016.

Year	Treatments	Kernel number		Ear number (m^−2^)	Ear Length (mm)	Ear Diameter (mm)	TKW (g)	Grain Yield (kg ha^−1^)
		(No. Ear^−1^)	(No. m^−2^)					
2015	Seed Soaking							
	CK1	411.9 ± 6.28c	3363.6 ± 44.01c	8.2 ± 0.10	14.0 ± 0.29d	42.3 ± 1.21c	254.6 ± 12.19c	6525 ± 262.21d
	S1	486.9 ± 18.33b	4011.5 ± 190.12b	8.2 ± 0.13	15.6 ± 0.56c	44.8 ± 1.41b	302.1 ± 2.40bc	7509 ± 194.00c
	S2	552.9 ± 24.80a	4566.0 ± 287.54a	8.3 ± 0.15	18.4 ± 0.45a	49.5 ± 0.66a	352.3 ± 7.60b	9702 ± 368.73a
	S3	495.6 ± 14.80b	4131.1 ± 177.24b	8.3 ± 0.12	17.0 ± 0.60b	48.2 ± 1.01a	323.4 ± 9.45a	8805 ± 326.90b
	Seed dressing							
	CK2	405.3 ± 15.98d	3338.1 ± 188.44d	8.2 ± 0.05	14.2 ± 0.12c	41.6 ± 1.06d	266.5 ± 13.10d	6385 ± 517.98d
	D1	437.9 ± 14.63c	3612.5 ± 115.63c	8.3 ± 0.07	15.6 ± 0.36b	43.9 ± 0.92c	282.8 ± 12.44c	7182 ± 439.21c
	D2	463.7 ± 15.01b	3840.5 ± 123.81b	8.3 ± 0.10	17.4 ± 0.56a	46.2 ± 1.24b	301.3 ± 9.38b	8089 ± 474.90b
	D3	493.8 ± 13.79a	4078.2 ± 175.02a	8.3 ± 0.14	18.1 ± 0.40a	48.5 ± 0.72a	329.6 ± 4.22a	8851 ± 400.66a
2016	Seed Soaking							
	CK1	417.7 ± 15.04c	3399.3 ± 101.43c	8.1 ± 0.05	13.3 ± 0.49c	40.4 ± 1.26d	262.6 ± 8.70d	6334 ± 303.49d
	S1	469.0 ± 9.54b	3861.1 ± 58.74b	8.2 ± 0.29	15.4 ± 1.03b	46.4 ± 0.95c	296.3 ± 10.55c	7778 ± 332.15c
	S2	507.7 ± 14.84a	4192.3 ± 168.19a	8.3 ± 0.14	17.5 ± 0.43a	51.7 ± 0.78a	346.5 ± 10.49a	11044 ± 463.98a
	S3	480.0 ± 7.55b	3960.7 ± 83.68b	8.3 ± 0.04	15.9 ± 0.37b	48.9 ± 1.20b	322.9 ± 17.76b	9956 ± 416.67b
	Seed dressing							
	CK2	431.7 ± 18.15c	3550.0 ± 120.64c	8.2 ± 0.16	13.7 ± 0.46c	43.0 ± 0.68c	259.1 ± 11.53c	5903 ± 410.85d
	D1	470.0 ± 13.11b	3878.1 ± 68.97b	8.3 ± 0.13	14.3 ± 0.40bc	46.3 ± 1.99b	296.9 ± 7.56b	7585 ± 334.51c
	D2	489.7 ± 6.03ab	4066.1 ± 84.52ab	8.3 ± 0.10	15.1 ± 0.42b	48.1 ± 0.53b	313.7 ± 7.80b	8288 ± 172.98b
	D3	516.3 ± 21.59a	4254.4 ± 171.65a	8.2 ± 0.05	17.0 ± 0.80a	50.7 ± 0.69a	335.1 ± 7.27a	8988 ± 137.15a

^*^TKW, thousand kernel weight; CK1, S1, S2, and S3 represents seed-soaking with paclobutrazol at the rate of 0, 200, 300, and 400 mg L^−1^, respectively. CK2, D1, D2, and D3 represent seed-dressing with paclobutrazol at the rate of 0, 1.5, 2.5, 3.5, g Kg^−1^ seed, respectively. Data are expressed as the mean ± S.D. (n = 3). Means followed by different letters within a column are significantly different at P < 0.05 as determined by the LSD test.

### Correlation analysis  of root characteristics and grain yield

**Figure 7:**
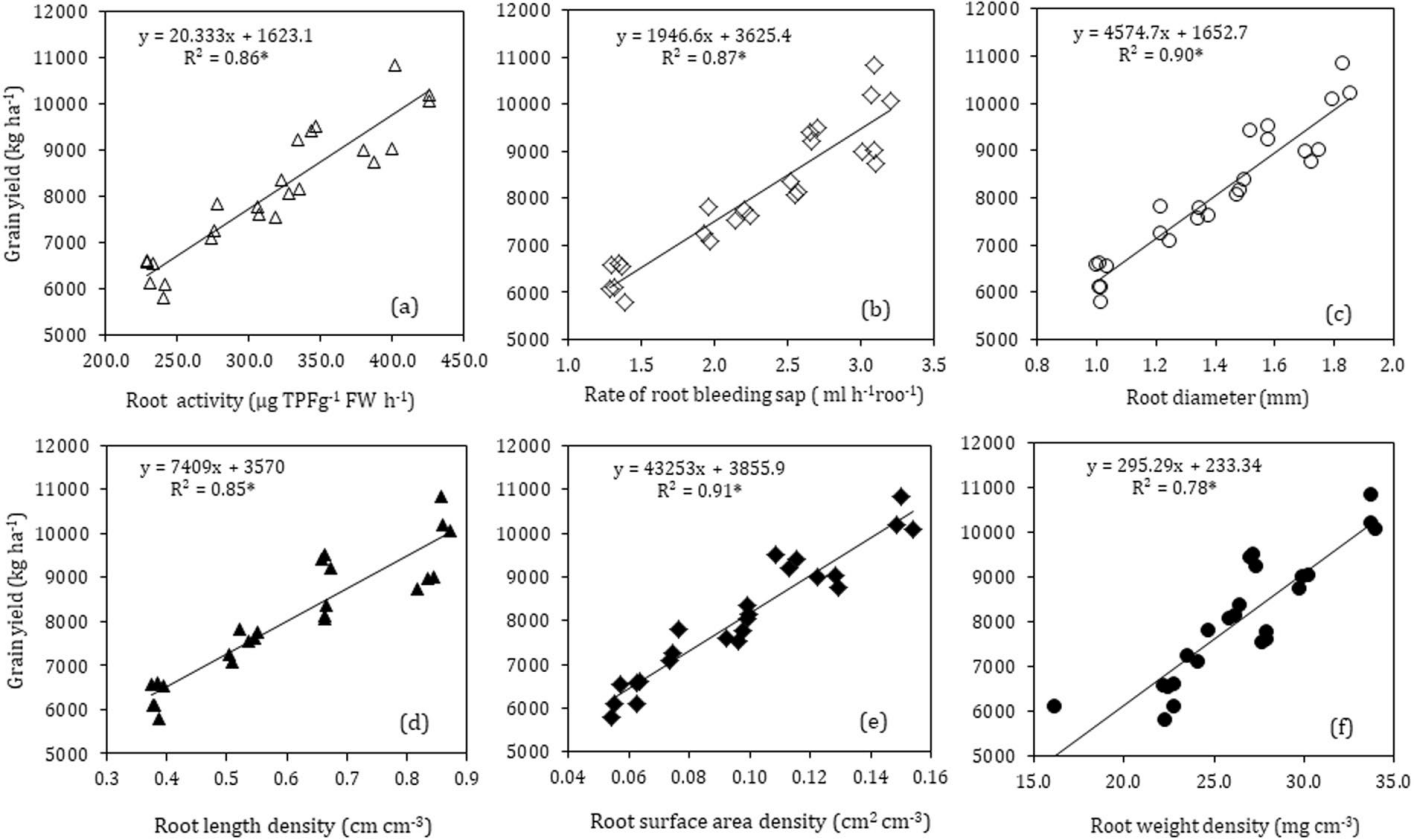
The relationship between the grain yield and root activity (**a**); root-bleeding sap flow (**b**); root diameter (**c**); root length density (**d**); root surface area density (**e**); and root length density (**f**).

Simple Pearson’s correlation analysis was used to determine the relationship between root growth characteristics and grain yield of maize (Fig. 7). The correlation analysis revealed a strong significant and positive correlation of root activity, the rate of root bleeding sap and root diameter with grain yield (Fig. 7a–c). Also, the results showed that RWD, RLD, and RSD were highly significantly correlated with maize grain yield (Fig. 7d–f).

## Discussion

### Effect of paclobutrazol on root growth and distribution

Maize growth and development in semi-arid farming areas of the Loess Plateau in China are solely dependent on precipitation water. Because of the limited and erratic rainfall pattern, the crop is often subjected to drought stress at various intervals during the growing season, severely affecting crop growth and yield. Roots are the primary organ for crop water and nutrient acquisition, and root spread and distributions had a substantial impact on plants growth and grain yield^10,33,34^. Previous research studies have revealed the efficacy of various triazoles, and paclobutrazol has been proved to be a potential anti-gibberellic triazole, could be used for canopy management of crops and thus as anti-lodging agents^27,35,36^. They are often referred as multi-stress protectants due to their innate potential of mitigating the negative effects of abiotic stresses had on plant growth and development, by regulating endogenous hormones level, enzymatic and non-enzymatic antioxidants and osmolytes^28,37–39^. However, there is a lack of scientific literature on how different concentration of paclobutrazol and application methods could affect maize roots growth and development across the root zones in the field. Therefore, in the present study, we determined the detailed root growth patterns across the root zones of maize to fill the research gap. The two years results of our field experiments showed that root activity and root-bleeding sap flow were significantly higher in paclobutrazol treatments than that in control treatment. Paclobutrazol, regardless of its concentration and application method significantly increased root activity and root-bleeding sap flow, and the best results were observed in S2 and D3 treatments. The root activity and bleeding sap flow increased with growth stages, and reached its peak at the silking stage and decreased after that. As root-bleeding sap is the manifestation of root pressure, therefore, the improved root-bleeding sap is attributed to higher root growth and root vigor in response to the paclobutrazol application. The study of Morita *et al*.^40^ showed the presence of a close relationship between the bleeding rate and the root traits in maize. The rate of root bleeding sap is correlated to active water absorption of the root system and reflects the physiological root activity^41,42^. Previously, Zhou and Ye^43^ and Yan *et al*.^44^ observed that uniconazole, a triazole with a function similar to paclobutrazol promoted root activity, root bleeding sap and improved root growth in crops. Zhao *et al*.^45^ also observed a higher root activity in rice and wheat treated with plant growth regulators.

Root diameter has been shown to have a significant influence on the ability of roots to penetrate into the soil^11^, extract water and nutrient in deep soil layers and translocate to aerial parts of the plant. A research study demonstrated that root length density, root weight density, root volume density, and the root diameter of crops are positively related to soil water availability^46^. In the present study, the root diameter of maize crop increased gradually from V7 to V9 stage, rapidly from V12 to R1 and decreased after that to the R6 stage, and was significantly higher in paclobutrazol treatments than untreated control, at all growth stages in 2015 and 2016. The increased root diameter is associated with larger cortical parenchyma or increased in the number of rows and diameter of cortical cells, as earlier demonstrated in maize and soybean treated with PGRs^28,47,48^. Nevertheless, our results are inconsistent with those of Khalil and Rahman^49^, who observed reduced shoot as well as roots growth in paclobutrazol treated maize seedlings. The possible explanation could be the different growth conditions. Earlier studies showed triazoles had  varied effects, from enhancement to inhibition effects on plant growth, depending on plant species, growing conditions, and concentration of the triazole used^27,32^. Primarily, triazoles treatments are associated with increased root extension but also promotes radial cell expansion^50^. The enhanced growth characteristics of roots are the ultimate consequence of radial cell expansion and association to the larger parenchyma cells^28^.

Root length density (RLD) and root surface area density (RSD) are pertinent determinants for characterizing crops root systems^9,34^. Root growth across the root zone has an immense influence on the potential capability for crop water and nutrients acquisition^51^. Strong relationships exist between the RLD and water and nutrients acquisition for crops^52^. Root surface area is becoming a major controlling factor for determination of uptake of relatively immobile nutrients such as P, to some extent K, and most of the micronutrients^10^. The limitation of root growing space in maize greatly affected root growth and decreased TTC reducing amounts and root absorbing area^53^. Our data clearly demonstrated that paclobutrazol treated plants have a greater RLD and RSD both at silking and physiological maturity, which increased with the increase of the concentration of paclobutrazol, except for higher concentration (S3) in seed soaking, which reduced the RLD and RSD, and are compatible with previous research studies^32,35,54^. The most significant differences for seed soaking treatments were observed mostly in 0–80 cm and for 0–70 cm soil profile in seed dressing treatments, at silking and physiological maturity, respectively. Though the RLD and RSD in 70–100 cm were slightly higher in paclobutrazol treatment, statistically no significant differences were observed when compared with CK. Soil water and nutrients availability in 0–60 cm soil profile are considered to be the most favorable and accessible for corn roots growth and development^9^, and root growth promotion and development by paclobutrazol treatments in the following soil depths could enhance crop’s potential capacity for nutrients and water uptake. The increased RLD and RSD in paclobutrazol treatments are directly attributed to improved root length, volume and other traits of maize root system by the paclobutrazol application. Our results are in line with previous studies, that the application of plant growth regulators increased root length, root volume, dry matter, and diameter in rape and tomato seedlings^23,55^. Moreover, triazoles are reported to inhibit gibberellic acid and enhance the levels of endogenous cytokinins and ABA^26,28^, which could be one of the possible reason for increased root length in paclobutrazol treated plants. Previous studies reported increased root length in triadimefon treated C. *roseus and* P. *fluorescens*^56,57^, associated with increased levels of endogenous cytokinin^31^. In addition, the stimulation of root growth may also be the consequence of increased assimilates partitioning towards the roots due to decreased demand by the shoots, as triazole reduces shoot elongation of plants, which are in agreement with the previous studies^23,27,58^. This suggests that paclobutrazol treated maize plants with an increased and fine root system would guarantee to explore and exploits soil for nutrients and water uptake, subsequently would increase the grain yield. Our correlation analysis revealed significant and positive relation of RLD and RSD with final grain yield of maize.

Moreover, paclobutrazol treatments significantly increased root dry weight, root/shoot ratio and greater RWD at most of the soil depths in 0–100 cm soil profile at different sampling stages, compared to that of untreated control plants. The root dry weight and root/shoot ratio of maize crop was considerably higher in S2 and D3 treatments during consecutive growing seasons (2015–2016). Banon *et al*.^59^ and Gu *et al*.^60^ observed a similar increasing trend of root weight and root to shoot ratio with paclobutrazol treated *P. angustifolia* and DCPTA treated maize seedlings. The effects of triazole on root growth vary from enhancement to inhibition, but almost in all cases resulted in an increased root/shoot ratio^61,62^. Our data showed that majority of the maize roots were in the 0–40 cm soil profile and the root weight density (RWD) tended to decreased substantially with the soil depth. Paclobutrazol application significantly promoted RWD of maize plants mostly in the 0–80 cm under seed soaking, and 0–60 cm under seed dressing treatments. The improved RWD is linked with the increased assimilate partitioning towards the roots rather than shoot, due to the reduction in shoot elongation by paclobutrazol, resulting in improved root architecture and greater root dry weight. Earlier reported by Qiu *et al*.^23^, uniconazole treatments significantly promoted root characteristics, including increased root length, volume and dry weight in rape seedlings. Our results are in line with the previous research in which paclobutrazol retarded shoot growth and promoted lateral root growth with more extensive root system in soybean^63^ and in wheat^64^. A significant and positive (R^2^ = 0.91**) correlation was observed between RSD and grain yield.

### Effect of paclobutrazol on ear characteristics and grain yield of maize

Crop growth and grain yield are closely related to the spread of roots, which determines the uptake and utilization of water and nutrients^52^. Zhang *et al*.^65^ reported that greater root biomass is significantly and positively correlated with ear characteristics and enhanced biomass and grain yields. Our results also showed that root growth and development under paclobutrazol application significantly (P < 0.05) improved ear characteristics and grain yield under both application methods, at each concentration, in both years, but at a different amount. This increase in the grain yield is attributed partly to decreased investment in above ground parts, due to a relatively stouter canopy of paclobutrazol treated plants, as well as enhanced grain filling in the treated plants due to the improved rooting system, which possibly increased the nutrients and water uptake. Triazole are reported to increase the photosynthetic efficiency, chlorophyll pigments, increase the leaf greenness, and root length in certain agronomic and horticultural crops^28,31,64^. The correlation analysis revealed significant and positive correlations between grain yield and root morphological traits. Interestingly, root activity, root-bleeding sap flow, RLD, RWDS and root dry weight of maize reached to the maximum values in S2 and D3 treatments under seed-soaking and dressing, respectively, and subsequently decreased in S3 treatment under seed-soaking. This decrease might be due to the inhibitory effect of paclobutrazol at higher concentration under seed-soaking. Therefore, an appropriate concentration for respective application method should be considered while paclobutrazol application in the maize crop. From our results, we assumed that for higher grain yield, a crop should also have higher root activity and greater root surface area, which would help crop plants to efficiently absorb and translocate nutrients from the deep soil in an efficient way. A crop with deeper root system could explore a large volume of soil, and with the large surface area or fine root system would more efficiently exploit a limited soil volume^52^. Nevertheless, roots spatial distribution and size may not be the limiting factor for certain nutrients acquisition (e.g., nitrogen and certain others due to their mobility in soil). However, under limited soil water and nutrient in arable land (especially dry-land farming), deeper and better root distribution would more proficiently utilize the limited water and heterogeneously distributed nutrients and would enhance crop growth and final grain yield.

## Conclusion

In summary, the RLD, RSD, and RWD showed evident difference almost in 0–70 cm under seed-soaking treatments, and in 0–60 cm soil profile under seed-dressing treatments. However, no significant differences were observed in RLD, RSD, and RWD in deeper soil profile below 80 under seed-soaking and 60 cm in seed-dressing treatments. The S2 (paclobutrazol at rate of 300 mg L^−1^ water) and D3 (paclobutrazol at rate of 3.5 mg kg^−1^ seeds) treated plants exhibited a larger root system and markedly increased the root activity, rate of root-bleeding sap flow, root diameter, root dry weight, and root/shoot ratio at each growth stage in both growing seasons, which allows roots to explore more volume of soil, subsequently improved ear characteristics and the final grain yield of maize.

## Methods

### Site description

**Figure 8:**
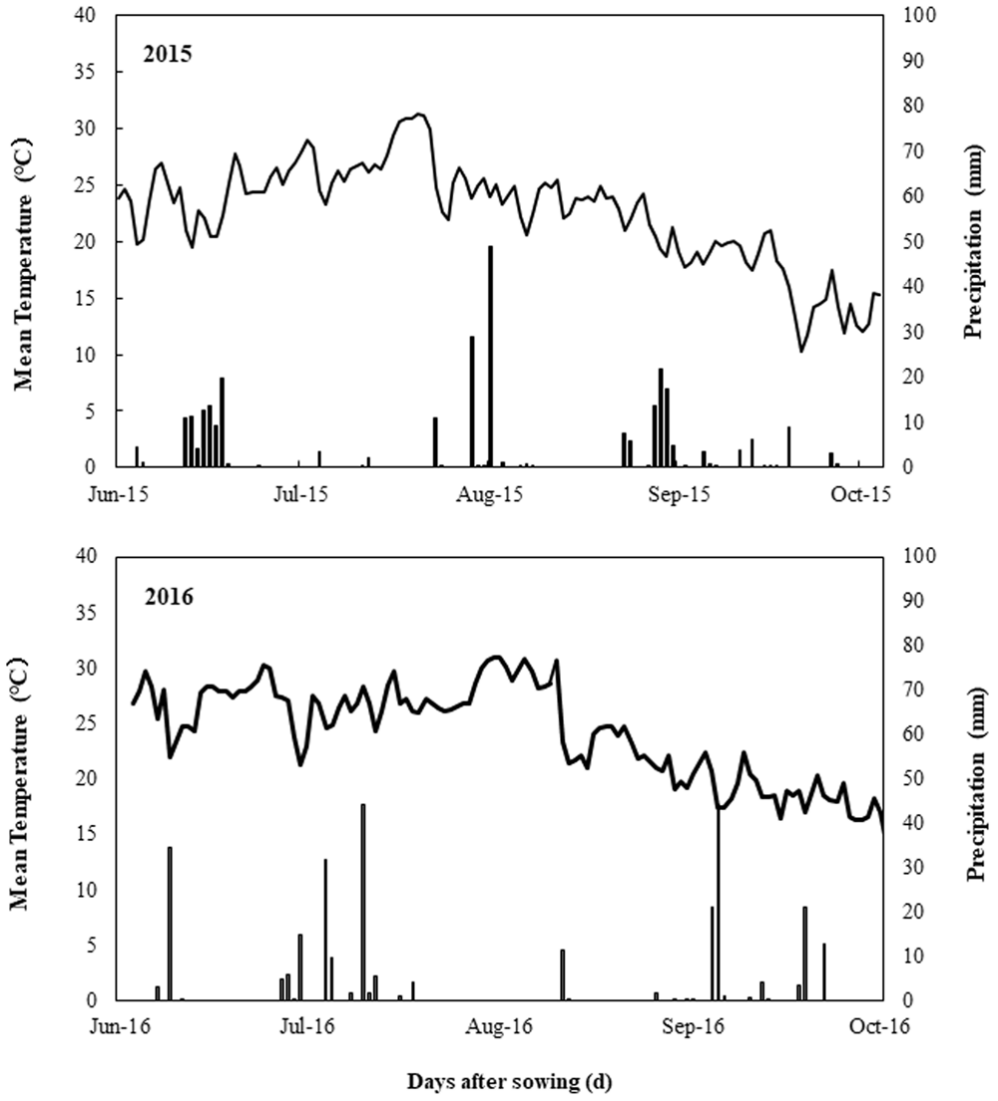
Mean temperature and rainfall during the growing seasons in 2015 and 2016.

Field experiments were conducted at the experimental farms of Northwest A&F University at Yangling (34°20′N, 108°04′E; 466.7 m above sea level), Shaanxi Province in 2015 and 2016. The experimental farms are located at the plateau of the Weihe plain at the north slope of Qinling Mountains, characterized by semi-arid and warm temperate drought-prone climate. The precipitation and air temperature during the experiment were measured by an automatic weather station at the experiment site (Fig. 8). The annual mean temperature ranges from 12~14 °C, with an extreme lowest temperature of −21~−15 °C. The average annual sunshine duration is about 2196 h with annual frost-free 190 days. Average annual precipitation is 581 mm. The soil of the experimental site is Heilutu, belonging to Cumuli-Ustic Isohumosols (light silt loam; according to Chinese Soil Taxonomy). The total N, P, and K concentrations in the top 20 cm were 1.43, 0.79 and 5.86 g kg^−1^, while the readily available N, P, and K concentrations were 57.81, 26.09 and 96.84 mg kg^−1^, respectively.

### Seed treatment and experimental design

Zhengdan (ZD 958), a summer maize variety was used in the experiment. The experiment comprised of two different application methods (seed-soaking and seed-dressing) with three different concentrations of paclobutrazol, each including a control treatment (CK). The concentrations of paclobutrazol were established in previous experiments. The seeds were first surface sterilized by immersing in a 3% solution of sodium hypochlorite for at least 20 minutes followed by washing several times with distilled water. For seed soaking, the maize seeds were primed at the rate 0, 200, 300 and 400 mg paclobutrazol L^−1^ water for 12 hrs. For seed dressing the seed were surface dressed with paclobutrazol at 0, 1.5, 2.5 and 3.5 g Kg^−1^ of seeds. Before sowing, seeds were dried at room temperature until moisture content reached to 10%. The hybrid seeds were manually sown on 14th June 2015 and 16th June 2016, while harvested on 11th October 2015 and 14th October 2016, respectively. Prior to planting, flood Irrigation was applied through a plastic pipe (50 mm diameter) to ensure germination. The experiment was laid out in Randomized complete block (RCB) design with three replications. The plot size was kept as 35 m^2^ (7 × 5 m) with the plant-to-plant and row-to-row distances of 20 cm and 60 cm, respectively. Recommended nitrogen fertilizer at the rate 240 kg N ha^−1^ was applied and urea (N46%) was used as N fertilizer. Half of the N fertilizer was manually applied prior to sowing. The remaining half was applied before the tasseling stage. A total of 150 kg phosphorus (P_2_O_5_) ha^−1^ as calcium superphosphate (P_2_O_5_ 12%) and 150 kg potassium (K_2_O) ha^−1^ as potassium sulfate (K_2_O 45%) was applied at sowing. The crop was solely dependent on soil moisture and natural rainfall in the season.

## Sampling and Measurement

### Root sampling

Roots samples were collected from each plot at the seventh leaf (V7), ninth leaf (V9), twelfth leaf (V12), silking (R1), and physiological maturity (R6) stages. At each sampling time, soil cores were taken by using a soil auger (diameter 8 cm) at three different locations i.e. at the plant spot, intra-plants and intra-rows spots. Each core was obtained from 0 to100 cm depth of the soil profile, each with 10 cm increment i.e., 0–10, 10–20, 20–30, 30–40, 40–50, 50–60, 60–70, 70–80, 80–90, and 90–100 cm. The soil cores were soaked in a plastic container overnight, and roots were stirred and sieved through a mesh (400 holes cm^−2^). The soil cores were then carefully washed by swirling water through it. The soil material and old dead roots debris were manually separated from the live roots^52^. The roots images from each soil core were obtained by using a digital scanner (Epson Perfection V700, Indonesia). The root images were then analyzed for root length, diameter and related root traits using WinRHIZO version 5.0 software (Regent Instruments Inc., Quebec City, Canada). The root length density (RLD, cm root cm^−3^ soil) and root surface area density (RSD, cm^2^ root cm^−3^ soil) were calculated according to Mosaddeghi *et al*.^66^ and Guan *et al*.^9^ The root dry weights were determined after drying the root samples in an oven at 105 °C for 30 min, and then at 75 °C until constant dry weight. The root weight density (RWD, mg root cm^−3^ soil) was calculated by dividing the root dry weight (mg) at a specific soil depth by the volume of the corresponding core sampling core (cm^3^), following Qi *et al*.^52^.

### Root activity, and collection of root-bleeding sap

Root vigor was measured according to the triphenyl tetrazolium chloride (TTC) method. Fresh roots were washed thoroughly with distilled water and cut into small pieces of 3–4 mm. A 0.5 g root sample was placed in graduated glass tubes containing 5 mL of 0.4% TTC solution and 5 mL of 0.1 mol L^−1^ phosphate buffer (pH 7.0) at 37 °C for 3 h. Then 2 ml of sulfuric acid (H_2_SO_4_) was added to the tubes to terminate the chemical reaction. The root activity was expressed by the amount of TPF (triphenyl formazan) deoxidized by TTC^52^.

The collection of root-sap was carried out according to Guan *et al*.^9^. Five maize plants were sampled were at V7, V9 and V12, R1 and R6 stage. The plants were cut at 10–12 cm above the soil surface at evening. A plastic film with cotton was placed on the upper end of the stalk, and a rubber band was used to ensure that plastic film was fixed firmly to the stalk. The bleeding sap in the flask was collected for 12 h, and its volume was measured. The delivery rate was expressed as concentration per unit time per root (ml h^−1^ root^−1^).

### Shoot biomass

Plants were sampled at V7, V9, V12, R1, and R6 stages. The aboveground plant parts were collected and separated into leaves, stems (including stem, sheath, and tassel) and ears (after anthesis). Dry matter of each component was determined after drying it in an oven at 105 °C for half an hour and then at 70 °C until to a constant weight.

### Ear characteristics and grain yield

Prior to physiological maturity, Ear m^−2^ were recorded in each treatment plot. At physiological maturity, thirty representative plants were randomly harvested in each plot (except the border plants). Ear length (cm), diameter (mm) and yield components such as grain weight (g) ear^−1^, kernels ear^−1^ were determined. Grain yield and thousand kernel weight (TKW) were converted to yield using a fixed grain water content of 12%.

### Data analysis

The data were subjected to one-way ANOVA by using SPSS software (SPSS Version 20.0, SPSS Inc., USA). The differences in means were compared by using the using Fisher’s protected least significant difference (LSD) test at 0.05% probability level. Simple Pearson’s correlations analysis was used to determine the relationship between root characteristics and grain yield of maize.
